# Luminal Microbes Promote Monocyte–Stem Cell Interactions Across a Healthy Colonic Epithelium

**DOI:** 10.4049/jimmunol.1301497

**Published:** 2014-06-06

**Authors:** Dagmara A. Skoczek, Petr Walczysko, Nikki Horn, Alyson Parris, Simon Clare, Mark R. Williams, Anastasia Sobolewski

**Affiliations:** * Gut Health and Food Safety Institute Strategic Program, Institute of Food Research, Norwich, Norfolk NR4 7UA, United Kingdom;; † School of Biological Sciences, University of East Anglia, Norwich, Norfolk NR4 7TJ, United Kingdom; and; ‡ Wellcome Trust Sanger Institute, Wellcome Trust Genome Campus, Cambridge, Cambridgeshire CB10 1SA, United Kingdom

## Abstract

The intestinal epithelium forms a vital barrier between luminal microbes and the underlying mucosal immune system. Epithelial barrier function is maintained by continuous renewal of the epithelium and is pivotal for gut homeostasis. Breaching of the barrier causes mobilization of immune cells to promote epithelial restitution. However, it is not known whether microbes at the luminal surface of a healthy epithelial barrier influence immune cell mobilization to modulate tissue homeostasis. Using a mouse colonic mucosal explant model, we demonstrate that close proximity of luminal microbes to a healthy, intact epithelium results in rapid mucus secretion and movement of Ly6C^+^7/4^+^ monocytes closer to epithelial stem cells. These early events are driven by the epithelial MyD88-signaling pathway and result in increased crypt cell proliferation and intestinal stem cell number. Over time, stem cell number and monocyte–crypt stem cell juxtapositioning return to homeostatic levels observed in vivo. We also demonstrate that reduced numbers of tissue Ly6C^+^ monocytes can suppress Lgr5EGFP^+^ stem cell expression in vivo and abrogate the response to luminal microbes ex vivo. The functional link between monocyte recruitment and increased crypt cell proliferation was further confirmed using a crypt–monocyte coculture model. This work demonstrates that the healthy gut epithelium mediates communication between luminal bacteria and monocytes, and monocytes can modulate crypt stem cell number and promote crypt cell proliferation to help maintain gut homeostasis.

## Introduction

The intestinal epithelium forms a vital barrier between the commensal microbes in the gut lumen and the underlying mucosal immune system. Barrier function is maintained by the constant renewal of the epithelium, which is driven by LGR5^+^ stem cells (1) located at the base of epithelial invaginations called crypts. On exiting the niche, stem cells give rise to progenitors, which proliferate and differentiate while migrating along the crypt axis until they are shed from the surface epithelium into the crypt lumen. Although the key factors required for epithelial renewal and regeneration in vitro have been identified (2, 3), the potential for modulation of this renewal by other cellular compartments exists (4, 5). Previous work showed that breach of the epithelial barrier exposes lamina propria immune cells to commensal bacteria, which triggers an innate immune response. This loss of barrier function was shown to cause mobilization of immune cells to specific sites at the epithelium (6), promoting regeneration of the epithelial barrier (7, 8). However, it is not known whether similar epithelial–immune cell interactions can occur during homeostasis (i.e., when the immune cells do not come into direct contact with commensal bacteria) (9). This raises a key question: Can the healthy epithelium mediate communication between luminal bacteria and immune cells and, in doing so, modulate its own renewal to maintain homeostasis?

Renewal of the intestinal epithelium is known to be under the influence of the gut microflora (e.g., germ-free mice have shorter crypts and a thinner mucus layer than do conventionally reared mice) (10). The crypt epithelium is equipped with pattern recognition receptors (11, 12), and emerging evidence suggests that the apical surface of epithelial cells can sense luminal microbes (13–15). Furthermore, specific commensal bacteria also were described to reside in close proximity to the apical surface of the colonic crypt epithelium during homeostasis (16). Commensal bacteria can come into closer contact with the epithelium through microbiota-induced alterations in the mucus layer, as can occur with different dietary components, dehydration, or antibiotics (17). This increasing body of evidence begs the question as to whether microbes acting at the apical surface of the intact epithelium can stimulate immune cell recruitment, tissue renewal, and mucus secretion as part of a localized “homeostatic” innate immune response.

Thus far, evidence has shown that lamina propria immune cells require a loss of epithelial barrier function and direct exposure to bacteria to mount an innate immune response. Therefore, much of what is known about immune–epithelial interactions comes from injury or infection studies. Seminal work has highlighted the importance of the spatial and temporal interactions between epithelial and immune cells during injury/infection. Chieppa et al. (6) first demonstrated that an immune cell can sample the gut lumen by extending processes between epithelial cells, and others investigators showed that, following injury, certain immune cells can relocalize to specific epithelial sites to bring about epithelial regeneration (7, 8, 18–20). Taken together, these findings suggest that different niche environments along the epithelial crypt axis can fine tune or modulate epithelial renewal during injury/infection. However, it is not known whether the healthy epithelium is permissive or can transduce microbial luminal inputs to subepithelial immune cells, which, in turn, regulates its own renewal.

To address this question, we developed a colonic mucosal explant model, which contains all of the cellular constituents of the colon and the potential to alter the mucus layer. By culturing mucosal explants at air–apical interface, we were able to maintain a polarized, physiological system. We developed a strategy whereby the luminal contents of the colon and part of the mucus layer were removed without compromising epithelial barrier function. *Escherichia coli*, LPS, or muramyl dipeptide (MDP) was added back to alter the luminal environment, allowing the acute spatial and temporal characteristics of innate immune cell mobilization within the colonic mucosa to be studied. In parallel, a similar model was recently developed (21).

Our findings demonstrate that, following luminal microbial input at the healthy apical surface of the colonic epithelium, there is an increase in epithelial proliferation. Recruitment of Ly6C^+^ 7/4^+^ monocytes into juxtaposition with the stem cell niche is required for the proliferative response and is recapitulated in a monocyte–colonic crypt coculture model.

## Materials and Methods

### Mice

LGR5-EGFP-Ires-CreERT2 and C57BL/6 mice (The Jackson Laboratory), MyD88-deficient mice and MyD88 villin cre mice (Welcome Trust Sanger Institute), and CX3CR1-GFP mice (kind gift from Claudio Nicoletti, Institute of Food Research) were used at age 8–12 wk. Generation and genotyping of the LGR5-EGFP-Ires-CreERT2 allele were described previously (1). All animal experiments were conducted in accordance with the Home Office Animals (Scientific procedures) Act of 1986, with approval of the University of East Anglia Ethical Review Committee, Norwich, U.K. and under Home Office project license number 80/1964.

#### Acute model of dextran sodium sulfate-induced colitis.

Distal colon was obtained from C57BL/6 mice with free access to 2.5% dextran sodium sulfate (DSS) in their drinking water for 7 d, with fresh solution replaced after 3 d. Mice were euthanized on day 7.

For the assessment of the role of monocytes in the response to luminal microbes, Lgr5EGFP mice were injected i.p. with 0.5 mg anti-mouse Ly6C Ab or IgG2a isotype control (both from Bio X Cell) on day 0. Significant monocyte depletion was confirmed by counting the number of Ly6C-expressing cells in the colonic mucosa on day 2.

### Human tissue

The human study was performed in accordance with approval from the East of England National Research Ethics Committee (LREC 97/124), and informed consent was obtained from all subjects. Colorectal tissue samples were obtained at rectosigmoid endoscopy from the sigmoid colon.

### Reagents

BrdU was purchased from Invitrogen, LPS was from Sigma, MDP was from Bachem, and TRITC-dextran (155 kDa) and Alexa Fluor 594–conjugated LPS (10 kDa) were from Molecular Probes.

#### Crypt culture media and supplements.

Advanced DMEM/F12, GlutaMAX, B27, and N2 were purchased from Invitrogen. Mouse recombinant growth factors used for mouse colonic crypt culture; murine recombinant epidermal growth factor and noggin (PeproTech), mouse recombinant R-spondin 1 (R&D Systems). Human recombinant growth factors used for human colonic crypt culture experiments were insulin growth factor-1 (Sigma), R-spondin 1 (Sino Biological or R&D Systems), Wnt 3A (R&D Systems), and Noggin (PeproTech). Matrigel Matrix, Growth-Factor Reduced was purchased from VWR International.

#### Immunolabeling.

Primary and secondary Abs used for immunolabeling: mouse anti-Ki67 Ab (Dako), rat anti-BrdU (Abcam), mouse anti-Muc2 and rabbit anti-chromogranin A (Santa Cruz), rabbit anti–caspase-3 (Cell Signaling); rabbit anti–E-cadherin (BD Bioscience); rat or rabbit F4/80, rat anti-7/4 (Abcam); rat or rabbit anti-Ly6C, Ly6G, and Gr-1 (BD Pharmingen); rabbit anti-CD11b; and rat IgG and rabbit IgG (Abcam). Immmunolabeling was visualized using an appropriate combination of species-specific Alexa Fluor–conjugated secondary Abs (488, 568, and 647 nm) raised in mouse, donkey, or goat (Invitrogen).

### Mucosal explant culture and immunofluorescent staining

Mouse distal colon was flushed with PBS to remove gut luminal contents and placed in culture media (Advanced DMEM/F12 and l-glutamine). The tissue was cut into 10-mm^2^ pieces, placed apical-side up on a Transwell insert (Corning-Costar), and put into a well containing culture media in a 12-well plate. Mucosal explants were stimulated at the apical surface with 1 μl LPS or culture media– or LPS-coated Affi-Gel beads, LPS, MDP, or *E. coli* (wild-type strain K12) diluted in culture media (Supplemental Fig. 1A, 1B). Mucosal explants were cultured at air–apical interface for up to 8 h in 95% oxygen inside an incubation chamber (Billups Rothenburg) at 37°C. At the end of the culture period, explants were fixed in 4% paraformaldehyde, frozen in isopentane, and stored at −20°C. Eight 20-μm cryosections (Leica CM 1100C cryostat) were permeabilized with 0.5% Triton-X (Sigma), blocked with 10% FBS, and stained with primary Abs diluted (1:100) in PBS overnight at 4°C, followed by the corresponding secondary fluorescence-conjugated Abs (1:200 in PBS) for 2 h. The sections were imaged using a confocal microscope (Zeiss 510 META) and an ×40 1.3 NA oil-immersion objective or an ×63 1.4 0.75-mm WD oil-immersion objective.

For studies of the mucus layer, mucosal explants were cultured for up to 1 h in the presence or absence of mCherry *E. coli* and fixed at 15-min intervals with methanol-Carnoy fixative, as previously described (22). For studies on immune cell localization, primary Abs to different innate immune cell subtypes were used (7/4^+^, Gr-1^+^, F4/80^+^). Immune cells were counted at the base, supra-base, mid, or top regions of the crypt (Supplemental Fig. 1D).

For in vitro proliferation experiments; BrdU (1 μM) was added to all culture media. Epithelial barrier function was assessed ex vivo by adding fluorescent TRITC-dextran (100 μM, 155 kDa) or Alexa Fluor 594–LPS (10 kDa) to the apical surface of mucosal explants cultured at the air–apical interface for up to 4 h and imaged by multiphoton microscopy.

### *E. coli* K12 fluorescent cloning and culture

#### Media, growth conditions, and transformations.

*E. coli* strains were grown in Luria–Bertani medium at 37°C. For the maintenance of plasmids, ampicillin was added to culture media at a concentration of 200 mg/ml. The *E. coli* strain K12 MG1655 (ATCC 700926; F-λ-ilvG-rfb-50rph-1) was transformed by electroporation using a Gene Pulser II (Bio-Rad, Hemel Hempstead, U.K.).

#### Fluorescently tagged and nonfluorescent *E. coli*.

The *E. coli* strain MG1655 was transformed with control DNA pUC18 (23) or plasmid DNA pmCherry (Clontech/Takara Bio Europe) encoding fluorescence protein mCherry to create strain pGH046. For fluorescence-based experiments, 20-ml cultures of *E. coli* were grown for 16 h in 250-ml flasks with shaking, and the cells were harvested by centrifugation at 4000 × *g* for 10 min at 18°C. Cell pellets were washed once with PBS before adjusting the cell suspension to give an OD 600 of 2.33, equivalent to a bacterial density of 1 × 10^9^ CFU/ml.

### Crypt isolation and culture

Colonic crypts were isolated from the distal colon of C57BL6 or LGR5EGFP mice, as previously described (2, 24, 25). The mouse colon was opened longitudinally; cut in small pieces; washed with PBS; incubated with HEPES-buffered saline: NaCl 140 mM, KCl 5 mM, HEPES, *d*-glucose 5.5 mM, Na_2_HPO_4_ 1 mM, MgCl2 0.5 mM, CaCl2 1 mM; and placed in HEPES-buffered saline that was devoid of both Ca^2+^ and Mg^2+^, and supplemented with EDTA (1 mM), for 1 h at room temperature. Crypts were liberated by serial rounds of vigorous shaking, crypt sedimentation, and collection. A total of 50–100 crypts was embedded in a 200-μl droplet of Matrigel Matrix, Growth Factor Reduced (VWR International) and seeded on No. 0 coverslips (VWR International) contained within a 12-well plate (Nunc). After polymerization at 37°C for 5–10 min, crypts were flooded with 0.5 ml mouse colonic crypt culture medium (advanced F12/DMEM containing B27, N2, *n*-acetylcysteine [1 mM], HEPES [10 mM], penicillin/streptomycin [100 U/ml], GlutaMAX [2 mM], epidermal growth factor [50 ng/ml], Wnt-3A [100 ng/ml], Noggin [100 ng/ml] [all from PeproTech] and R-spondin 1 [1 μg/ml; R&D Systems]). For the human study, colonic crypts were isolated from biopsies from patients undergoing colonoscopy, as previously described (24, 25), seeded in Matrigel (26), and flooded with human colonic crypt culture medium (advanced F12/DMEM containing B27, N2, *n*-acetylcysteine [1 mM], HEPES [10 mM], penicillin/streptomycin [100 U/ml], l-glutamine [2 mM], Wnt-3A [100 ng/ml], insulin-like growth factor-1 [50 ng/ml], Noggin [100 ng/ml], R-spondin 1 [500 ng/ml], and the ALK 4/5/7 inhibitor A83-01-01 [0.5 μM]) (26). MDP and LPS were used at a concentration of 0.1 μg/ml for crypt-proliferation studies (Fig. 2C).

### Bone marrow–derived monocyte isolation

Bone marrow–differentiated monocytes were isolated and cultured as previously described (27). Briefly, bone marrow cell suspensions were isolated by flushing femurs and tibias of 8–12-wk-old C57BL6 mice (Charles River, Sulzfeld, Germany) with complete RPMI 1640 (+10% FCS, +1% Pen/Strep [Invitrogen]). Cell suspensions were passed through a 70-μm nylon web and seeded at a concentration of 10^6^ cells/ml onto six-well ultra-low attachment surface plates (Corning Costar). Cells were supplemented with 20 ng/ml rmM-CSF (ProSpec) and cultured in a humidified incubator at 37°C and 5% CO_2_. Following 5 d in culture, the nonadherent monocyte-enriched population (>99% viability via Trypan Blue exclusion) was removed and placed in coculture with colonic crypts at different cell concentrations.

### Crypt–monocyte coculture

For monocyte–crypt coculture experiments, freshly isolated bone marrow–derived monocytes were resuspended at different concentrations (25 × 10^3^–1 × 10^6^) in Matrigel Matrix, Growth Factor-Reduced containing colonic crypts and cultured for 2 d in mouse crypt culture media as stated above. For the human study, colonic crypts were isolated from biopsies obtained from patients undergoing colonoscopy, as previously described (2, 24, 25, 28), and cocultured with the monocytic cell line THP-1 (ATCC #TIB-202), as described above, for 2 d in a humidified incubator (5% CO_2_, 37°C). For proliferation studies, BrdU (1 μM) was added after 24 h in culture; 8 h later, cocultures were fixed in 4% paraformaldehyde and stained with Abs to BrdU or Ki67, as described above (3, 26). The numbers of BrdU^+^ or Ki67^+^ nuclei in the equatorial plane of the crypts were counted and expressed as a percentage of the total number of crypt nuclei stained with DAPI (VECTASHIELD). The percentages of BrdU^+^ or Ki67^+^ nuclei were plotted against the number of mouse monocytes or THP-1 cells cocultured with mouse or human colonic crypts, respectively.

### Confocal and multiphoton microscopy

Following immunofluorescent staining, samples were visualized using laser scanning confocal (Zeiss 510 META), multiphoton (LaVision BioTec), or epifluorescence (Nikon Ti) microscopy. An ×63 (1.4 NA) objective was used to obtain confocal images, and image stacks were taken at 1–3-μm intervals, which allowed selection of precise focal planes.

Multiphoton microscopy was used to visualize the localization of fluorescent dextran (TRITC-dextran) in mucosal explants. An ×40 water-immersion objective (1.15 NA) was used to capture images from sequential planes that were acquired in z dimensions (1 μm each) to form a z stack. Each plane represents an image of 512 × 512 μm in the *x* and *y* dimensions. An excitation wavelength of 740 nm was used to visualize tissue autofluorescence, and an optical parametric oscillator wavelength of 1100 nm was used to visualize fluorescence of TRITC-dextran, Alexa Fluor 594–LPS, or mCherry *E. coli*.

### Image analysis

The crypt hierarchy was divided into four regions along the crypt axis, each containing an equal number of nuclei and designated base, supra-base, mid, and top, in addition to a submucosa and muscle region located below the mucosa. In some cases, the crypt was divided into three equal regions: base, mid, and top. The percentage of cells (or nuclei) positive for a specific marker (e.g., 7/4, BrdU) was determined for each region (Supplemental Fig. 1D). Morphometric analysis was performed using ImageJ software. Three-dimensional images were acquired with multiphoton microscopy and rendered in Volocity (Improvision).

### Statistics

Experiments were performed at least three times. Data are expressed as mean ± SE, and significance was determined by one-way ANOVA with post hoc Tukey analysis. The *p* values < 0.05 were considered significant.

## Results

### LPS and *E. coli* treatment of an intact barrier promotes crypt cell proliferation

**FIGURE 1.**
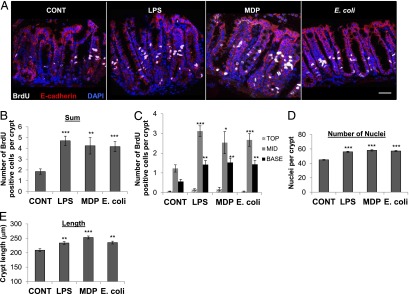
*E. coli* and LPS induce proliferation of the colonic epithelium at the stem cell niche and the transit-amplifying zone ex vivo. (**A**) Representative confocal images of BrdU (white), E-cadherin (red), and nuclei/DAPI (blue) immunolabeling of the colonic mucosa following 4 h of ex vivo culture with apical LPS, MDP, or *E. coli* stimulation. Scale bar, 50 μm. (**B**) Total BrdU incorporation ex vivo is increased in LPS-, MDP-, or *E. coli*–treated mucosal explants (*n* = 3). (**C**) BrdU incorporation is increased compared with control in the crypt base (*n* = 3) and the mid-crypt region (*n* = 3) following 4 h of luminal stimulation with LPS, MDP, or *E. coli*. Morphometric analysis of LPS-, MDP-, or *E. coli*–treated mucosal explants showed a significant increase in the number of nuclei/crypt (*n* = 5) (**D**) and the length of colonic crypts (*n* = 3) (**E**) compared with control explants. *p < 0.05, **p < 0.01, ***p < 0.001.

**FIGURE 2.**
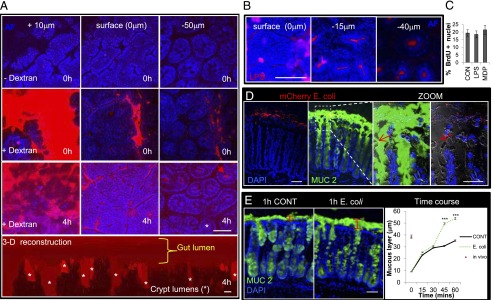
Intact epithelial barrier function and increased mucus secretion in the presence of mCherry *E. coli* during mucosal explant culture. (**A**) Representative immunofluorescent multiphoton images visualizing TRITC-dextran (red) and autofluorescence of the epithelium (blue) in mucosal explant culture. Images acquired at a plane 10 μm above the surface epithelium showed dextran present in the gut lumen at 0 and 4 h of mucosal explant culture. Images at the surface (0 μm) showed an intact epithelium, with no dextran observed between the epithelial cells. Fifty microns below (−50 μm) the surface epithelium, dextran was restricted to the lumens of colonic crypts (red arrow), also shown in the three-dimensional reconstruction of TRITC-dextran distribution in the mucosal explant following 4 h in culture (*bottom panel*). Asterisks denote crypt lumens in cross section and three-dimensional reconstruction. Data are representative of *n* = 4 independent experiments. (**B**) Representative immunofluorescent multiphoton images showing fluorescent Alexa Fluor 594–LPS (red) at the surface and −15 and −40 μm below the surface epithelium (blue) confined to the crypt lumens following 4 h of mucosal explant culture. Data are representative of *n* = 3 independent experiments. (**C**) Bar graphs showing no effect of LPS or MDP, added to the basal compartment, on colonic crypt cell BrdU incorporation (*n* = 3). (**D**) Representative immunofluorescent multiphoton images of MUC2 (green) expression in mucosal explants cultured for 1 h in the presence of mCherry *E. coli* (red). Zoom shows mCherry *E. coli* bacteria (red arrows) in close proximity to and above the surface epithelium. Experiments were repeated three times. (**E**) Representative immunofluorescent multiphoton images of MUC2 (green) expression in mucosal explants cultured for 1 h in the presence or absence of *E. coli*. Graph (*right panel*) shows that apical *E. coli* stimulation causes a significant increase (*n* = 3) in mucus thickness following 45 min or 1 h of stimulation in mucosal explant culture compared with control-treated explants. Data are mean ± SEM. Scale bars, 50 μm. ***p < 0.001.

Apical/luminal stimulation of mucosal explants with fluorescent mCherry or nonfluorescent *E. coli*, MDP, or LPS for 4 h resulted in a significant increase in ex vivo BrdU incorporation of the colonic epithelium (i.e., E-cadherin^+^ cells, Fig. 1A, 1B). In particular, there was an increase in epithelial proliferation in the mid/transit amplifying zone and the base/stem cell niche region of the crypts compared with control-treated explants (Fig. 1C). Morphometric analysis showed that 4 h of *E. coli*, MDP, or LPS luminal stimulation resulted in an increase in the number of nuclei/crypt axis (Fig. 1D) and an increase in crypt length (Fig. 1E). E-cadherin labeling was similar after 4 h in explant culture (Fig. 1A), suggesting that the colonic epithelium remained intact. A live functional multiphoton imaging approach using fluorescent TRITC-dextran (Fig. 2A) and fluorescent LPS (Fig. 2B) confirmed the integrity of the epithelial barrier by demonstrating dextran and LPS localization only to the colonic lumen following 4 h in culture. Tissue autofluorescence also showed that the surface epithelium formed an intact layer, with no epithelial gaps during explant culture (Fig. 2A). Furthermore, basal membrane exposure of the colonic epithelium to LPS or MDP in colonic crypt culture had no effect on BrdU incorporation (Fig. 2C), which suggested that the increased proliferation in response to MDP or LPS in mucosal explant culture was not due to a direct effect on the epithelial basal membrane. In addition, the majority of fluorescent mCherry *E. coli* were located on top of the mucus layer, and none was detected beneath the surface epithelium (Fig. 2D). However, there were instances in which the bacteria were in close proximity to the luminal surface epithelium. Prior flushing of luminal contents removed the loosely adherent mucus layer and part of the firm inner mucus layer, which subsequently reformed during culture, approaching in vivo thickness (nonflushed) after 1 h (Fig. 2E), as shown previously (29). *E. coli* increased the rate and thickness of mucus layer formation, indicative of an innate immune response (Fig. 2E). The viability (assessed by activated caspase-3 labeling) of mucosal explants following 8 h of culture was similar to that observed in immediately fixed native colon (Supplemental Fig. 1C).

### Recruitment of Ly6C^+^7/4^+^ cells to the surface epithelium and the epithelial stem cell niche following luminal stimulation with LPS, MDP, or *E. coli*

**FIGURE 3.**
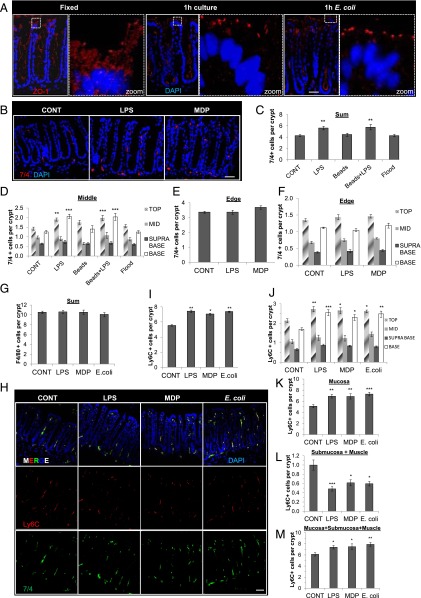
Localized recruitment of 7/4^+^/Ly6C^+^ cells to the surface epithelium and the stem cell niche following 1 h of apical stimulation of an intact epithelial barrier with LPS, MDP, or *E. coli.* (**A**) Representative immunofluorescent confocal images showing intact ZO-1 (red) following 1 h in mucosal explant culture. (**B**) Representative immunofluorescent images of 7/4^+^ cells (red) and nuclei/DAPI (blue) showing recruitment of 7/4^+^ cells in LPS- and MDP-treated (1-h) explants. (**C**) The addition of a 1-μl volume of LPS (0.01 μg/ml) or Affi-Gel Blue beads precoated with LPS to the apical surface of mucosal explants caused a significant increase in the number of 7/4^+^ immune cells in the colonic mucosa (*n* = 3) compared with control-treated beads only or flood-treated explants (20 μl volume) following 1 h in culture. (**D**) Significant recruitment of 7/4^+^ cells to the top and base epithelium following 1 h of apical stimulation with 1 μl of LPS or LPS-coated beads (*n* = 3). **p < 0.01, ***p < 0.001. No change in 7/4^+^ immune cell localization following apical stimulation with l μl of control media, beads alone (beads), or 20 μl of LPS (flood). Graphs showing 7/4^+^ cell recruitment at the middle (D) and lack of 7/4^+^ recruitment at the edge or edge of the mucosal explant (**E** and **F**) in response to central treatment with LPS or MDP (*n* = 3). (**G**) No change in the number of F4/80^+^ cells following LPS, MDP, or *E. coli* treatment (*n* = 7). (**H**) Representative immunofluorescent image showing double-positive (yellow/MERGE) Ly6C^+^ (red) 7/4^+^ (green) cells. Scale bar, 50 μm. Significant increase in the number of Ly6C^+^ immune cells in the colonic mucosa (*n* = 3) (**I**) and Ly6C^+^ cells at the top and base epithelium (**J**) following 1 h of apical stimulation with MDP, LPS, or *E. coli* (*n* = 3). An increased number of Ly6C^+^ immune cells in the colonic mucosa (**K**) resulted in a significant decrease in Ly6C^+^ cells in the submucosa and smooth muscle layers (*n* = 3) (**L**) and an increase in the total number of cells in the colonic explant tissue (*n* = 3) (**M**). *p < 0.05, **p < 0.01, ***p < 0.001. Data are mean ± SEM.

We next investigated the prospect that epithelial sensing of luminal microbes on one side of an intact epithelium led to the recruitment of immune cells on the other side of the epithelium. The incidence and distribution of several lamina propria immune cells (7/4^+^, F4/80^+^, Ly6C^+^) in explant culture were unchanged compared with their in vivo status (Supplemental Fig. 1E, 1G). However, following a 1-h luminal stimulation with LPS, there was an increase in the total number of 7/4^+^ cells in the mucosa and an increase in the numbers both at the crypt base/stem cell niche and surface crypt epithelium (Fig. 3) compared with control-treated mucosal explants. In addition to the exclusion of mCherry *E. coli*, fluorescent LPS, and dextran (Fig. 2B), we confirmed that the epithelium was intact via ZO-1 expression at apical tight junctions (Fig. 3A). The next series of important experiments demonstrated that the LPS stimulus was localized to the middle of the explant and did not spill over the edges into the well below (Supplemental Fig. 1B). 7/4^+^ cell mobilization occurred only in the middle of the explant at the origin of the stimulus and not at the explant edges (Fig. 3C–F), which is consistent with the localized central application of LPS-coated Affi-Gel beads.

**FIGURE 4.**
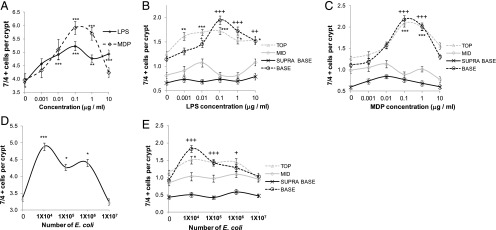
Concentration-dependent increase in 7/4^+^ cells in the colonic mucosa following 1 h of luminal stimulation with LPS, MDP, or *E. coli.* LPS caused a concentration-dependent increase in the total number of 7/4^+^ cells in the mucosa (**A**), whereas MDP (A) and *E. coli* (**D**) exhibited a dome-shaped concentration response curve (*n* = 3). LPS (**B**), MDP (**C**), and *E. coli* (**E**) caused a significant increase in the numbers of 7/4^+^ cells present in the top (*n* = 3) and base regions (*n* = 3) of the colonic mucosa compared with control-treated explants. Data are mean ± SEM. *p < 0.05, **p < 0.01, ***p < 0.001, ^+^*p* < 0.05, ^++^*p* < 0.01, ^+++^*p* < 0.001.

Colocalization studies using a series of immune cell markers showed that the majority of 7/4^+^ cells were also Ly6C^+^ (Fig. 3H) and displayed the same pattern of mobilization in response to LPS, MDP, and *E. coli* (Fig. 3H–J). No recruitment of F4/80^+^ cells occurred in response to luminal microbial stimulation (Fig. 3G), and only 12.4 ± 1.5% (*n* = 3, mean ± SEM) of Ly6C^+^ cells were F4/80^+^. To determine whether the increased number of Ly6C^+^ cells in the mucosa following LPS, MDP, or *E. coli* treatment originated from beneath the mucosa layer, we counted the number of Ly6C^+^ cells in the underlying submucosa and muscle layers. These experiments demonstrated that the significant increase in Ly6C^+^ cells in the mucosa (Fig. 3K) was associated with a significant reduction in the number of Ly6C^+^ cells in the submucosa and muscle layers (Fig. 3L) and an increase in the total number of Ly6C^+^ cells (Fig. 3M) following LPS, MDP, or E. *coli* treatment. The same mobilization profile occurred when using the Gr-1 marker (Supplemental Fig. 2A, 2B). The Gr-1 Ab recognizes the Ly6G and Ly6C Ags on neutrophils and monocytes, respectively, and the 7/4^+^ Ag is also expressed on both of these cell types (30–32). 7/4^+^ cells were positive for CD11b and negative for the neutrophil marker Ly6G, suggesting that the Ly6C^+^7/4^+^Gr-1^+^ cells were of a monocyte phenotype (Supplemental Fig. 2C, 2D). Although studies using explant tissue from CX3CR1GFP^+^ mice showed a significant increase in double-positive CX3CR1/Ly6C cells at the crypt base following 1 h of apical treatment with LPS, the numbers of these cells/crypt were low (Supplemental Fig. 2E, 2F), with <6% of Ly6C cells being positive for CX3CR1 (Supplemental Fig. 2G). The mobilization of 7/4^+^ cells to the crypt epithelium was concentration dependent in the case of LPS, whereas MDP and *E. coli* caused a dome-shaped response curve (Fig. 4A, 4D). At lower concentrations of LPS, MDP, or *E. coli*, 7/4^+^ cells were recruited in similar numbers to the mucosa; however, at higher concentrations, MDP and *E. coli* did not cause any net recruitment of 7/4^+^ cells into the mucosa. This perceived lack of response at higher concentrations may have been due to faster or slower 7/4^+^ migration rates that were not detected at this time point or it may reflect no migration at all. In any case, we chose the concentration of MDP, *E. coli*, and LPS that exerted maximal recruitment of 7/4^+^ cells into the mucosa at 1 h for all experiments in this study (0.1 μg/ml, 10^4^ bacteria, and 0.1 μg/ml, respectively).

### Rapid 7/4^+^ monocyte recruitment to the stem cell niche and surface epithelium

**FIGURE 5.**
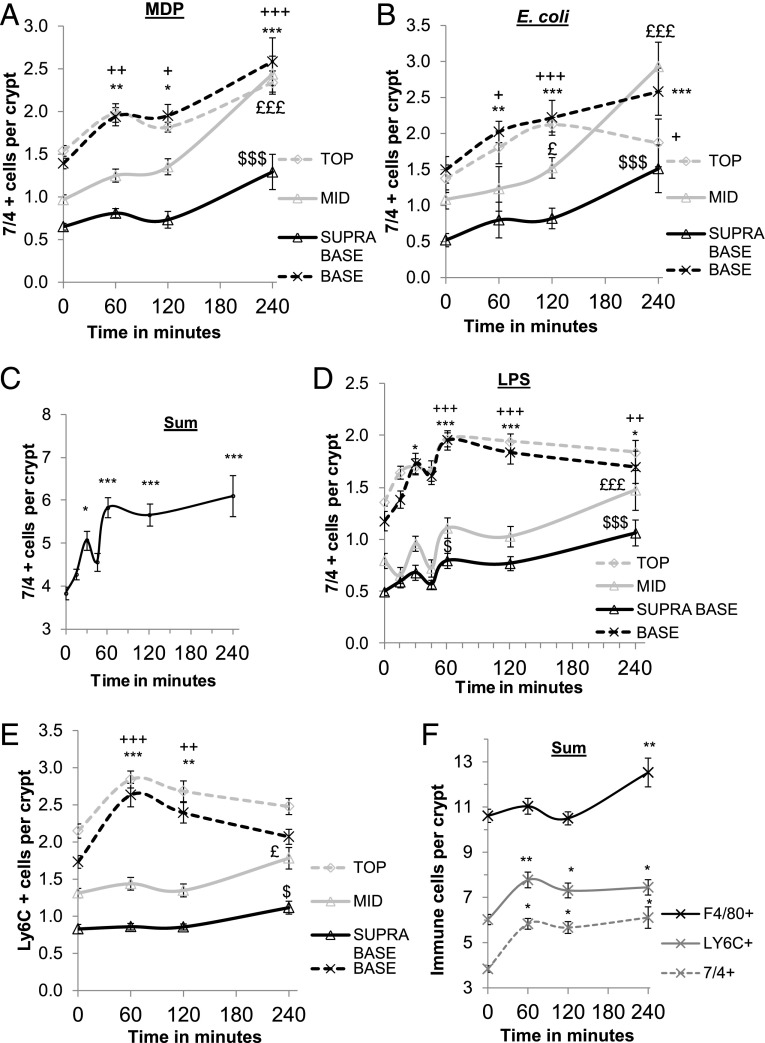
7/4^+^ cell recruitment to the stem cell niche occurs after 30 min of apical LPS stimulation. (**A** and **B**) Graphs showing a significant increase in 7/4^+^ cell recruitment to specific zones of the crypt epithelium over 240 min. After 60 min of *E. coli* (B) or MDP (A) treatment, there was a significant increase in the number of 7/4^+^ cells in the base (*n* = 3) and top regions (*n* = 3) of the crypt epithelium compared with control-treated explants. *^/+^*p* < 0.05, **^/++^*p* < 0.01. After 240 min, 7/4^+^ cells increased significantly in the mid and supra-base regions of the crypt (*n* = 3), ^£££/$$$^*p* < 0.001, and the base and top regions remained significantly increased (*^/+^*p* < 0.05, ***^/+++^*p* < 0.05). (**C**) Graph showing a significant increase in the total number of 7/4^+^ cells recruited to colonic mucosa after 30 min and 120 and 240 mins of LPS apical treatment (*n* = 3). *p < 0.05, ***p < 0.001. 7/4^+^ cell (**D**) and Ly6C^+^ cell (**E**) recruitment to specific zones of the crypt epithelium over 240 min. After 30 min, there was a significant increase in the number of 7/4^+^ cells in the base crypt epithelium (*n* = 3), whereas after 60 min, both the base and top epithelium had significantly more Ly6C^+^ or 7/4^+^ cells (*n* = 3) compared with control-treated explants. *p < 0.05, ***^/+++^*p* < 0.001. Ly6C^+^ cells decreased in the crypt base and top after 240 min, and both Ly6C^+^ and 7/4^+^ cells increased in the mid (*n* = 3) and supra-base (*n* = 3) regions of the crypt. ^£^*p* < 0.05, ^£££^*p* < 0.001, ^$^*p* < 0.05, ^$$$^*p* < 0.001. (**F**) The total number of F4/80^+^ cells increased after 240 min of apical LPS stimulation (*n* = 3). **p < 0.01. Data are mean ± SEM.

To map out the early spatial and temporal characteristics of microbial-induced 7/4^+^Ly6C^+^ cell mobilization to the crypt epithelium, we performed a time course study. 7/4^+^ immune cells were rapidly mobilized to the base and top crypt region 1 h after MDP and *E. coli* treatment (Fig. 5A, 5B). This significant increase in 7/4^+^ cells at the crypt base and top was still evident at 4 h poststimulation. Studies with LPS showed a significant increase in the total number of 7/4^+^ cells in the mucosa at 30 min, which was still significantly higher than control explants 4 h after treatment (Fig. 5C). The more detailed LPS time course also showed that significant 7/4^+^ cell recruitment to the base and top crypt regions occurred after 30 and 60 min, respectively, with mid and supra-base regions showing significant increases in 7/4^+^ cell numbers only after 4 h of LPS treatment (Fig. 5D). Mid and supra-base 7/4^+^ cell time courses were similar in MDP-, *E. coli*–, and LPS-treated explants (Fig. 5A, 5B, 5D). Similar patterns of mobilization were shown using the Ly6C^+^ marker (Fig. 5E). Of note, 7/4^+^ cells were never observed migrating through the epithelium into the lumen; they always resided beneath the basal crypt epithelial membrane. Interestingly, after 4 h of stimulation with LPS, the number of F4/80^+^ cells increased in the colonic mucosa (Fig. 5F).

### 7/4^+^/Ly6C^+^ monocyte recruitment and increased epithelial proliferation are abrogated in MyD88-deficient mice

**FIGURE 6.**
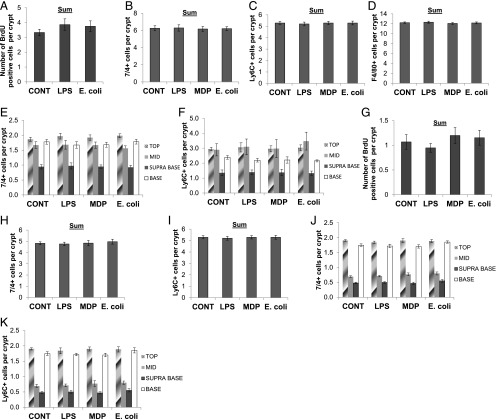
Increased epithelial proliferation in response to luminal microbial input is abrogated in MyD88-deficient mice, along with a lack of 7/4^+^ cell recruitment to the stem cell niche and surface epithelium. (**A**) Total BrdU incorporation in MyD88-deficient mice following luminal stimulation with LPS or *E. coli* was the same as in control-treated mucosal explants (*n* = 3). There was no change in 7/4^+^ (**B**), Ly6C^+^ (**C**), or F4/80^+^ (**D**) cell numbers in the colonic mucosa and a lack of recruitment to the base or top crypt epithelium (**E** and **F**) in MyD88-deficient mice (*n* = 3) following apical LPS, MDP, or *E. coli* treatment. (**G**) Total BrdU incorporation in villin cre MyD88–deficient mice following luminal stimulation with LPS, MDP, or *E. coli* was the same as in control-treated mucosal explants (*n* = 3). There was no change in 7/4^+^ (**H**) or Ly6C^+^ (**I**) cell numbers in the colonic mucosa, and there was a lack of recruitment to the base or top crypt epithelium (**J** and **K**) in villin cre MyD88–deficient mice (*n* = 3) following apical LPS, MDP, or *E. coli* treatment. Data are mean ± SEM.

To determine whether the proliferation and/or recruitment of 7/4^+^/Ly6C^+^ cells to the stem cell niche in response to LPS, MDP, and *E. coli* was through the MyD88-signaling pathway, we used MyD88-deficient and villin cre–MyD88–deficient mice (Wellcome Trust Sanger Institute). Epithelial proliferation in response to LPS or *E.* coli was abrogated in either MyD88-deficient mice (Fig. 6A) or villin cre–MyD88-deficient mice (Fig. 6G) ex vivo. Recruitment of 7/4^+^ or Ly6C^+^ cells to the colonic mucosa (Fig. 6B, 6C, 6H, 6I) or to the crypt base or surface epithelium in response to luminal LPS, MDP, or *E. coli* was also absent in mucosal explants cultured from both strains of mice (Fig. 6E, 6F, 6J, 6K). No change in F4/80^+^ cell recruitment was observed (Fig. 6D).

### 7/4^+^/Ly6C^+^ cells juxtapose directly with crypt epithelial LGR5EGFP^+^ stem cells

**FIGURE 7.**
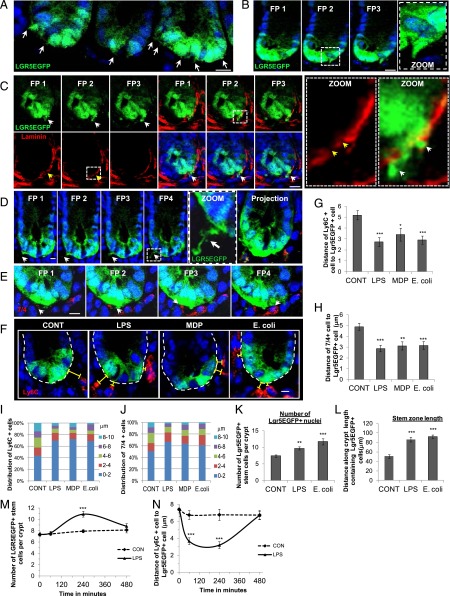
Apical microbial input causes juxtapositioning of Ly6C^+^/7/4^+^ cells with LGR5EGFP^+^ epithelial stem cells and increases the number of LGR5EGFP^+^ cells in mucosal explant culture. Representative examples of immunofluorescent confocal images of LGR5EGFP^+^ cell processes in freshly fixed/in vivo colonic tissue extending into the lamina propria (**A** and **B**) and extension of LGR5EGFP^+^ processes through gaps in laminin (red) in the basement membrane (**C**). White arrows denote cell processes; yellow arrows denote gaps in the basement membrane. White dashed-lined squares denote regions of interest displayed in the *insets*. Representative immunofluorescent confocal images of LGR5EGFP^+^ cell processes extending into the lamina propria and contacting immune cells (**D**) and reciprocal contact between 7/4^+^ (red) cell processes and LGR5EGFP^+^ (green) cell (**E**). (**F**) Ly6C^+^ cells moved closer to LGR5EGFP^+^ stem cells following apical *E. coli* and LPS treatment. Representative immunofluorescent confocal images of Ly6C^+^ cell (red) localization following LPS, MDP, or *E. coli* treatment. There were significantly shorter distances between Ly6C^+^ cells. White-dashed lines denote crypt bases. Yellow brackets denote distance between the LGR5EGFP^+^ cell and the Ly6C^+^ cell. (**G**) or 7/4^+^ cells (**H**) and LGR5EGFP^+^ stem cells following 1 h of LPS, MDP, or *E. coli* treatment (*n* = 3). Relative distribution of Ly6C^+^ cells (**I**) or 7/4^+^ cells (**J**) within a 10-μm boundary of the LGR5EGFP^+^ cell basal membrane. Morphometric analysis of *E. coli*– and LPS-treated mucosal explants in LGR5EGFP^+^ mice showed a significant increase in the number of LGR5EGFP^+^ nuclei (**K**) and a significant increase in the length of the LGR5EGFP zone (**L**) compared with control. (**M**) The numbers of LGR5EGFP^+^ cells increased significantly following 240 min of LPS treatment (*n* = 3), and they returned to control levels at 8 h. (**N**) Average distance between Ly6C^+^ cells and LGR5EGFP^+^ cells following 8-h treatment with LPS in mucosal explant culture; Ly6C^+^ cells were significantly closer to stem cells at 60 and 240 min after LPS treatment (*n* = 3). Scale bar, 10 μm. FP = focal plane images taken every 2 μm. Data are mean ± SEM. *n* = 3 *p < 0.05, **p < 0.01, ***p < 0.001, versus control-treated explants.

Recruitment of Ly6C^+^ monocytes to the crypt base (Figs. 3–5) raised the question of whether 7/4^+^/Ly6C^+^ cells were interacting with epithelial stem cells. LGR5-EGFP-Ires-CreERT2 mice were used, which express EGFP in the epithelial stem cells of the colon (1). *E. coli*, LPS, or MDP were used to apically stimulate mucosal explants from LGR5EGFP mice. Localization patterns for 7/4^+^, Gr-1^+^, and F4/80^+^ cells in response to luminal LPS and MDP in LGR5EGFP mice were the same as observed in C57BL6 mice (data not shown). Confocal imaging of LGR5EGFP^+^ stem cells showed the occasional presence of LGR5EGFP^+^ cell processes extending beneath the crypt base into the lamina propria in either in vivo control or LPS/MDP/*E. coli*–treated explants (Fig. 7A–E), and the processes were shown to extend through gaps in the laminin-rich basement membrane (Fig. 7C). Furthermore, these stem cell processes were generally associated with immune cells (Fig. 7D, 7E). There also were examples of reciprocal contact between 7/4^+^ cell processes and LGR5EGFP^+^ cells (Fig. 7E). Confocal image analysis showed that 7/4^+^/Ly6C^+^ cells moved closer to LGR5EGFP^+^ stem cells following luminal treatment with LPS, MDP, or *E. coli* (Fig. 7F–H); of those located within a 10-μm radius of the crypt base, a larger percentage was positioned closer to stem cells (Fig. 7I, 7J). Similar results were obtained with the Gr-1^+^ marker (Supplemental Fig. 3A, 3B). Using LGR5EGFP mice, we also demonstrated that 4 h of either LPS or *E. coli* treatment caused a significant increase in the number of LGR5EGFP^+^ stem cells/crypt and the length of the LGR5EGFP^+^ stem cell zone compared with control explants (Fig. 7K, 7L). We also demonstrated that, at later time points in culture (8 h), the number of LGR5EGFP^+^ stem cells, the distance between a crypt stem cell and a Ly6C^+^ cell (Fig. 7M, 7N), the number of crypt nuclei, and the crypt length all returned to control levels; interestingly, these corresponded with a return to in vivo levels for Ly6C^+^ cell number and distribution along the longitudinal crypt axis (Supplemental Fig. 3E, 3F).

### Monocytes stimulate proliferation of colonic crypts

**FIGURE 8.**
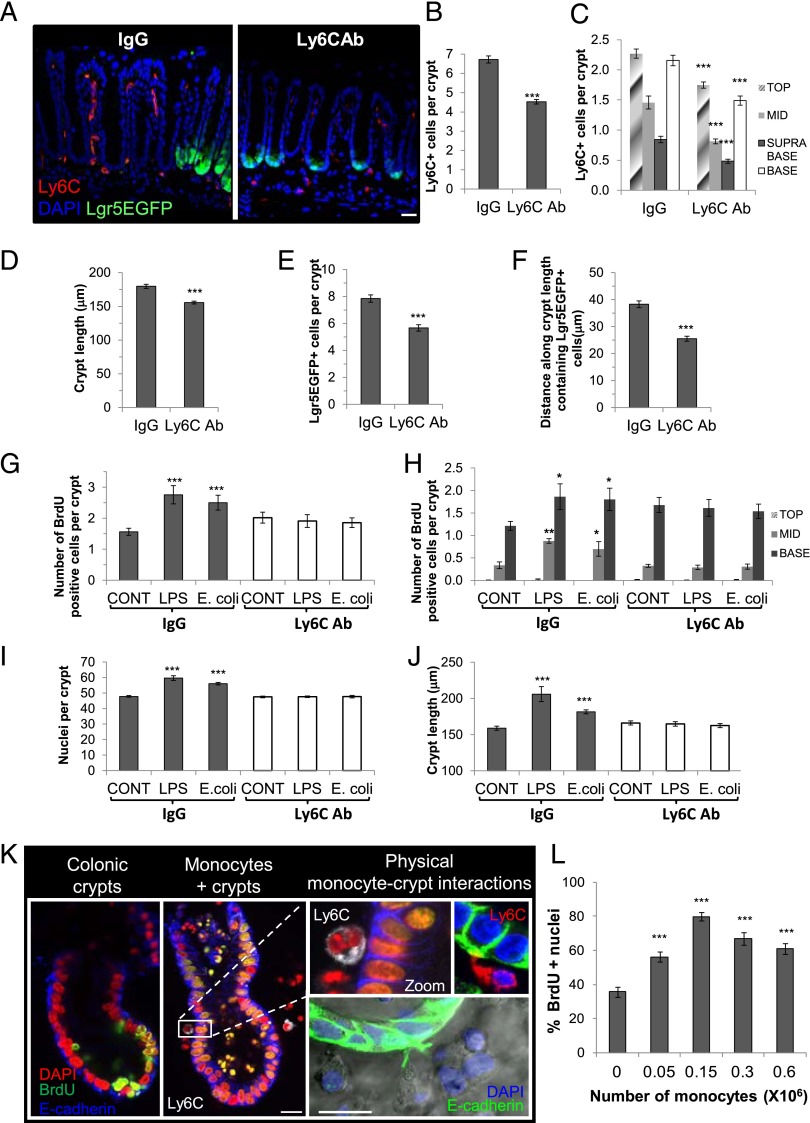
Monocytes induce proliferation of colonic crypts. (**A**) Representative confocal images of Lgr5EGFP colon from IgG and Ly6C Ab–treated mice. Scale bar, 50 μm. There was a significant reduction in Ly6C^+^ cells/crypt (*n* = 3) in the mucosa (**B**) and at all regions along the crypt axis (*n* = 3) (**C**) in Ly6C Ab–treated mice. Significant reductions were noted in the crypt length (**D**), number of Lgr5EGFP^+^ stem cells/crypt (**E**), and length of the stem cell zone (**F**) in the colon of Lgr5EGFP mice treated with an Ly6C-neutralizing Ab compared with IgG-treated mice (*n* = 3). LPS- or *E. coli*–stimulated mucosal explants from Ly6C Ab–treated mice showed no change in the number of BrdU^+^ cells/crypt (**G**) and no change in BrdU incorporation in each crypt region (**H**), crypt nuclei (**I**), or crypt length (**J**) in response to LPS compared with control. (**K**) Representative confocal images of BrdU incorporation (green) of colonic crypts in the presence or absence of monocytes showing juxtapositioning of a monocyte (white) to the crypt epithelium (E-cadherin blue). White-dashed line shows region of interest displayed in the *inset*. Images on the *right* show further examples of cell contacts of a Ly6C^+^ monocyte (red) with the crypt epithelium (E-cadherin green) and physical interaction of an epithelial process (E-cadherin positive-green) and monocytes in coculture. Scale bar, 10 μm. (**L**) Graph showing a monocyte-induced increase in BrdU incorporation of cultured colonic crypts (*n* = 3). *p < 0.05, **p < 0.01, ***p < 0.001.

These data suggested that Ly6C^+^ monocyte recruitment to the stem cell niche could play a role in promoting epithelial cell proliferation. We first embarked on an in vivo study in which we depleted monocytes in LGR5EGFP mice using a blocking Ab to the Ly6C Ag. Upon sacrifice 48 h after i.p. injection, the number of Ly6C^+^ cells in the mucosa was significantly depleted (Fig. 8A–C). There also were decreases in the crypt length, the number of LGR5EGFP^+^ stem cells/crypt, and the length of the stem cell zone in the colons of Ab-treated mice (Fig. 8D–F). Mucosal explant studies with this tissue demonstrated no increase in epithelial crypt BrdU incorporation, crypt nuclei, or crypt length following LPS or *E.* coli treatment in the explants cultured from the Ly6C Ab–treated mice, which was in contrast to explants cultured from IgG-treated mice (Fig. 8G–J).

With the aim of understanding the direct effect of monocytes on the colonic epithelium, we next developed a less complex ex vivo coculture model of these two cell types. There is a paucity of monocytes in mouse blood; therefore, to obtain sufficient numbers for experimentation, primary bone marrow–differentiated monocytes were isolated as previously described (27). This monocyte-enriched cell population was cocultured with colonic crypts. Confocal image analysis showed evidence of physical interactions/juxtapositioning of Ly6C^+^ cells with the colonic crypt epithelial cells during ex vivo coculture (Fig. 8K), as well as increased BrdU incorporation of the crypt epithelium in the presence of monocytes. Quantification of BrdU incorporation showed that this occurred in a cell density–dependent manner (Fig. 8L). The mitogenic effect of monocytes on the crypt epithelium also was evident using human colonic crypts cocultured with a THP-1 human cell line (Supplemental Fig. 4).

## Discussion

Previous work demonstrated that mobilization of specific immune cell subtypes to the crypt stem cell niche following injury causes epithelial regeneration (7, 8, 18), but whether luminal microbes can influence epithelial–immune cell interactions across a healthy epithelial barrier is unknown. We obtained several lines of evidence to demonstrate that the epithelial barrier in our colonic mucosal explant culture model was intact: the expression of junctional proteins ZO-1 and E-cadherin was unaltered, fluorescent *E. coli* added to the luminal side did not gain access to the lamina propria, multiphoton imaging of live explants showed no gaps in the surface epithelium using the tissue autofluorescence signal, and luminal TRITC-dextran and fluorescent LPS were excluded from the lamina propria. Confinement of luminal treatment to the apical surface was confirmed by application of microscopic beads and localization of the immune response to the region beneath the microbial stimulus. This polarized explant system was used to demonstrate that exposure of the intact, healthy epithelium to luminal microbes initiates a MyD88-dependent epithelial-driven scheme of events that promotes intestinal homeostasis. This scheme is characterized by increased mucus secretion, rapid (30 min) recruitment of monocytes to the crypt epithelium and juxtapositioning of monocytes with crypt stem cells, which is followed 4 h later by a monocyte-dependent increase in proliferation and stem cell number ex vivo. Monocyte–crypt stem cell juxtapositioning and increased stem cell number are transient and, at 8 h after luminal input, return to levels observed in vivo (i.e., restoration of homeostasis). Moreover, we also show that Ly6C^+^ monocytes help to maintain crypt stem cell number in vivo and describe the striking phenomenon whereby crypt stem cells can extend processes into the lamina propria to make physical contact with Ly6C^+^ monocytes. We also demonstrate that, in a reciprocal fashion, Ly6C^+^ monocytes can extend cellular processes to gain closer proximity to LGR5EGFP^+^ stem cells. An ex vivo monocyte–crypt coculture model demonstrated that monocytes exhibit similar physical interactions with the crypt and promoted an increase in proliferation of the crypt epithelium. These findings shed new light onto the complex network of microbial–epithelial–immune cell interactions that occur across the intact intestinal barrier to maintain tissue homeostasis.

Innate immune cell interactions are important for maintaining homeostasis and quickly resolving perturbations in the local gut environment, thus preventing the development of chronic inflammation. Central to maintaining intestinal homeostasis is the microbiota, which is protective against injury (11) and modulates epithelial crypt length (33–35). Commensal bacteria were shown to reside within the mucus layer in close proximity to the crypt epithelium, underpinning the potential for homeostatic communication (16). We speculate that a compromised mucus layer results in proximity of bacteria (and their products; e.g., MDP or LPS) at the surface epithelium. This triggers a localized recruitment of Ly6C^+^ monocytes to the underlying stem cell niche that promotes proliferation, epithelial turnover, and shedding of cells and any adherent bacteria at the surface epithelium (36). The increase in Ly6C^+^ monocyte number at the stem cell niche could provide a source for an increased local concentration of secreted factors that potentially modulate epithelial stem cell proliferation, differentiation, cell migration, and cell shedding and/or recruit other immune cells to the stem cell niche. It is also possible that the physical contacts made between monocytes and epithelial stem cells are required for modulation of stem cell activity, and the presence of a larger number of monocytes may also increase the frequency of these events. Restoration of homeostasis may involve Ly6C^+^ monocyte relocalization back to their homeostatic position that is further away from the epithelium, where monocyte–epithelial cell contact would be less frequent and/or local concentrations of monocyte-secreted factors would be less influential on crypt cell proliferation/renewal. Similar site-specific immune–epithelial interactions have been observed following injury or infection (6–8). The ligand(s) and receptors potentially involved in this process are the subject of ongoing work. The dependence of the concomitant increase in mucus production on immune cell recruitment is also under investigation.

In this study, a proportion of the Ly6C^+^ monocytes recruited to the epithelium originate from the submucosa and muscularis. Significant tissue pools of local monocytes exist in other systems (37) and may be important in the colon for rapid recruitment following changes in homeostasis and inflammation, in addition to circulating peripheral pools. Future work will use real-time intravital imaging to study the routes of monocyte recruitment to the crypt stem cell niche; this approach will also help to determine whether the dome-shaped response curves that we observed in response to luminal *E. coli* and MDP are due to differing migration rates or routes at higher or lower concentrations. Several chemokines were shown to recruit Ly6C^+^ monocytes in the intestine, and the epithelium was shown to express MCP-1 (38). Monocytes (Ly6C^+^/Ly6B^+^) are rapidly recruited to the site of infection during intestinal inflammation (39) and are generally considered to be inflammatory cells. Conversely, sufficient and timely infiltrations of Ly6C^+^ monocyte/macrophage subsets have been implicated in recovery after spinal cord injury (20), angiogenesis/tissue repair (19), protection against intestinal parasites (40), vascular homeostasis (41), and neuroprotection (28). In our study, the Ly6C marker is coincident with the 7/4/Ly6B Ag, which was shown to be a marker for recently generated macrophages with a more immature phenotype (42). Recent work showed that Ly6C^+^ cells are the precursors for either inflammatory or homeostatic macrophages or dendritic cells (43–46). We observed a return to baseline numbers of Ly6C^+^ cells after 8 h in culture with LPS (along with a concomitant return to crypt stem cell number and crypt length) in addition to an increase in F4/80^+^ cells, suggesting a possible switch from an inflammatory to a homeostatic phenotype, as was described previously (47). Whether these monocytes become homeostatic macrophages or dendritic cells in the longer term is the subject of ongoing work.

The MyD88-signaling pathway was shown to be required for monocyte recruitment in DSS-induced colitis (12). In our studies, we demonstrate that, under homeostatic conditions, the epithelial MyD88 pathway is also required for monocyte mobilization to the healthy crypt epithelium. In the current study, failure of monocyte recruitment to the stem cell niche following luminal addition of microbes in MyD88^−/−^ mice correlated with the absence of a proliferative response. This is similar to what was shown in a DSS model of colitis using MyD88^−/−^ mice, confirming that stem cell proliferation is also reduced (11). Interestingly, MDP-induced mobilization of Ly6C^+^ monocytes to the epithelium also was dependent on the epithelial MyD88 pathway. NOD2 signaling in colonic epithelial cells is important for maintaining gut homeostasis (48) and, more recently, it was shown to be required for host epithelial chemokine-mediated responses and dendritic cell recruitment following *Trichuris muris* infection (49). There is emerging evidence of cross-talk between the TLR- and NOD-signaling pathways that may explain this dependency (50). Of note, NOD2 stimulation by MDP in colonic epithelial cells enhances TLR-mediated epithelial barrier function and chemokine production (51); this raises the possibility that, in our study, low concentrations of TLR ligands exist at the luminal surface, and the introduction of MDP evokes enhanced TLR signaling with subsequent monocyte migration and crypt cell proliferation. This topic remains the subject of future work in the laboratory. MDP/NOD2 forms part of the protective innate immune response and was shown to recruit Ly6C^+^ cells in response to influenza A virus (52) or *Citrobacter rodentium* (53).

Our study demonstrates that a reduction in lamina propria monocytes significantly reduces the numbers of LGR5EGFP stem cells in vivo. In addition, we showed that the presence of luminal bacteria increased the number of LGR5EGFP^+^ stem cells ex vivo compared with control over a 4-h period, suggesting that microbes may help to support the maintenance of the epithelial stem cell niche. We also demonstrate a direct proproliferative effect of mouse monocytes on the colonic epithelium in a crypt–monocyte coculture model, which mimics in vitro the close proximity of immune cells to the crypt epithelium in vivo. Monocytes were shown to exert proliferative effects in a variety of systems (19, 54). Our coculture studies focused on the early effects of monocytes on the crypt epithelium (<2 d), over which time LGR5EGFP transgene expression is lost (55, 56). However, LGR5EGFP expression is restored at longer time points, which would allow an assessment of the effects of monocytes on crypt epithelial stem cell number in the longer term. Interestingly, other cells of myeloid origin also exerted similar mitogenic effects on the mouse colonic epithelium (data not shown), suggesting the potential for redundancy in the system. This effect was also evident using human colonic crypts cocultured with a monocyte cell line, suggesting that monocytes play a role in renewal of the human intestinal epithelium.

We demonstrate that the epithelial–MyD88 pathway is required to respond to luminal microbial inputs and maintain homeostasis; whether epithelial TLR activation (13, 14) or luminal sampling (6, 57) also play a role is the subject of future work. This study shows that, following luminal microbial input, Ly6C^+^ monocyte recruitment can have early profound effects on the intestinal epithelium, possibly through secretion of regenerative factors (e.g., Wnt ligands) (51) or through physical interactions, which could determine whether the ensuing immune response is proinflammatory or resolving. It is also possible that a reciprocal relationship between the crypt epithelium and monocytes occurs, whereby the colonic epithelium influences the differentiation of monocytes to a macrophage/dendritic cell phenotype through either physical interactions or via secreted factors, as has been seen in other systems (58–61). Understanding the reciprocal nature of epithelial–monocyte interactions in health versus disease promises valuable insights into intestinal homeostasis and pathogenesis.

## Supplementary Material

Data Supplement
