# Supplementary material for: Luminal Microbes Promote Monocyte–Stem Cell Interactions Across a Healthy Colonic Epithelium
Source: J Immunol. 2014 Jun 6;193(1):439–51. doi: 10.4049/jimmunol.1301497 (PMC4067517; doi:10.4049/jimmunol.1301497)
Supplement: Data Supplement [file supp_193_1_439__index.html]

Luminal Microbes Promote Monocyte–Stem Cell Interactions Across a Healthy Colonic Epithelium — Luminal Microbes Promote Monocyte–Stem Cell Interactions Across a Healthy Colonic Epithelium — Data Supplement 

# Luminal Microbes Promote Monocyte–Stem Cell Interactions Across a Healthy Colonic Epithelium

## Data Supplement

**Files in this Data Supplement:**

- Supplemental Figures 1 (PDF)
