# Supplementary material for: Luminal Microbes Promote Monocyte–Stem Cell Interactions Across a Healthy Colonic Epithelium
Source: J Immunol. 2014 Jun 6;193(1):439–51. doi: 10.4049/jimmunol.1301497 (PMC4067517; doi:10.4049/jimmunol.1301497)
Supplement: Data Supplement [file 1301497_JI_1301497_Supplemental_Figures_1.pdf]

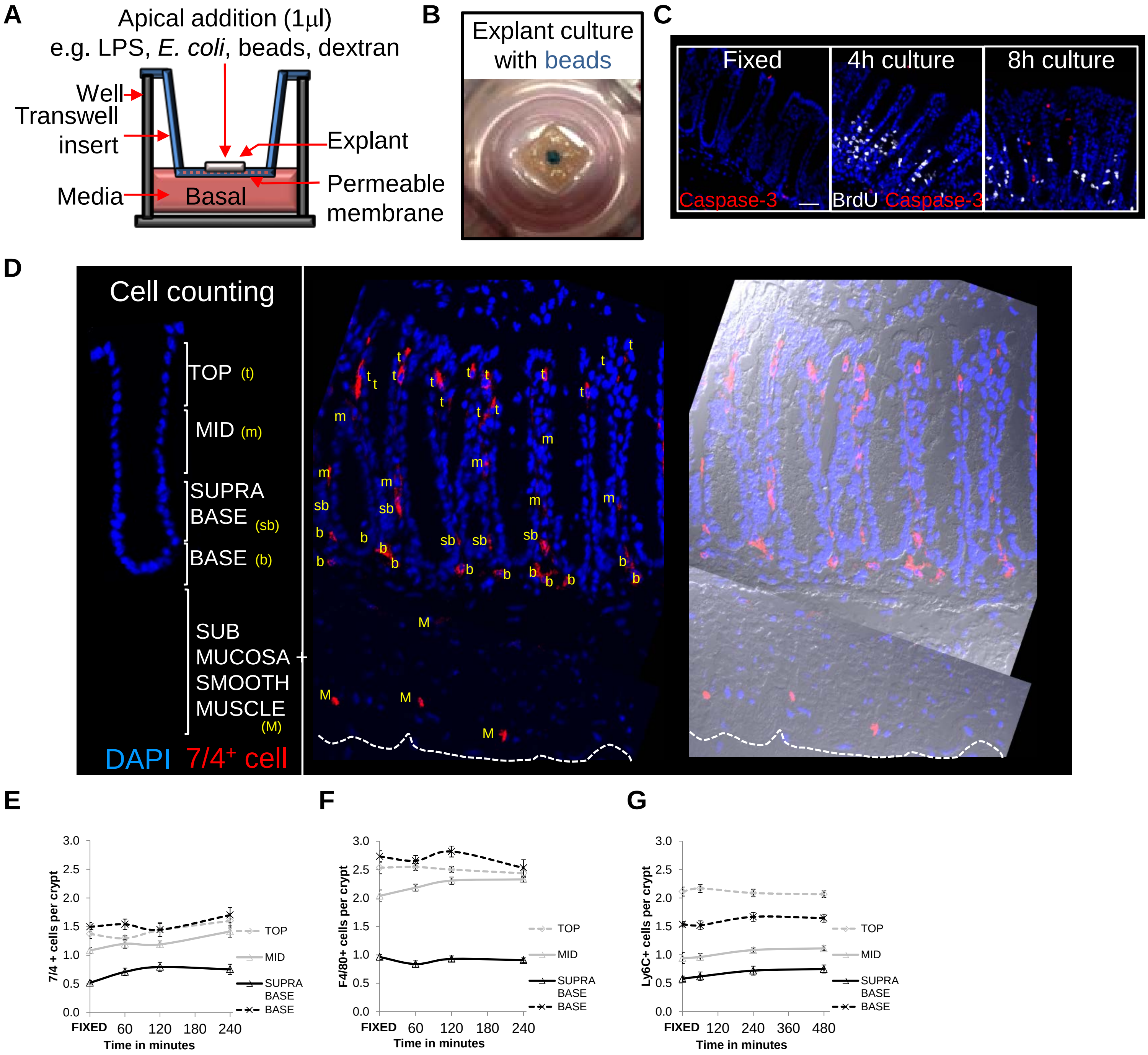

Supplementary Figure 1. Schematic showing colonic mucosal explant set-up, model validation and quantification of immune cell localisation in mucosal explant culture.
A Colonic mucosal explant schematic. **B** Image showing location of Affigel™ Blue beads in the middle of a mucosal explant. **C** Viability of mucosal explants following 4 and 8 hours culture at air-apical interface. Representative confocal images showing BrdU incorporation (white) and low caspase-3 activity (red) after 4 and 8 hours in culture is comparable to caspase 3 activity in vivo / fixed tissue. Nuclei (blue-DAPI). Data is representative of n=3 independent experiments. Scale bar 50μm. **D** Representative immunofluorescent image of 7/4$^+$ cells (red) and DAPI stained nuclei (blue) demonstrating counting strategy for immune cell localisation studies. Experiments were validated by the experimenter counting blind. **E-G** Timecourse graphs showing that the distribution of 7/4+ (**E**), Gr-1+ (**F**) and Ly6C+ (**G**) cells along the crypt axis remains unchanged with respect to *in vivo* /fixed tissue following mucosal explant culture.

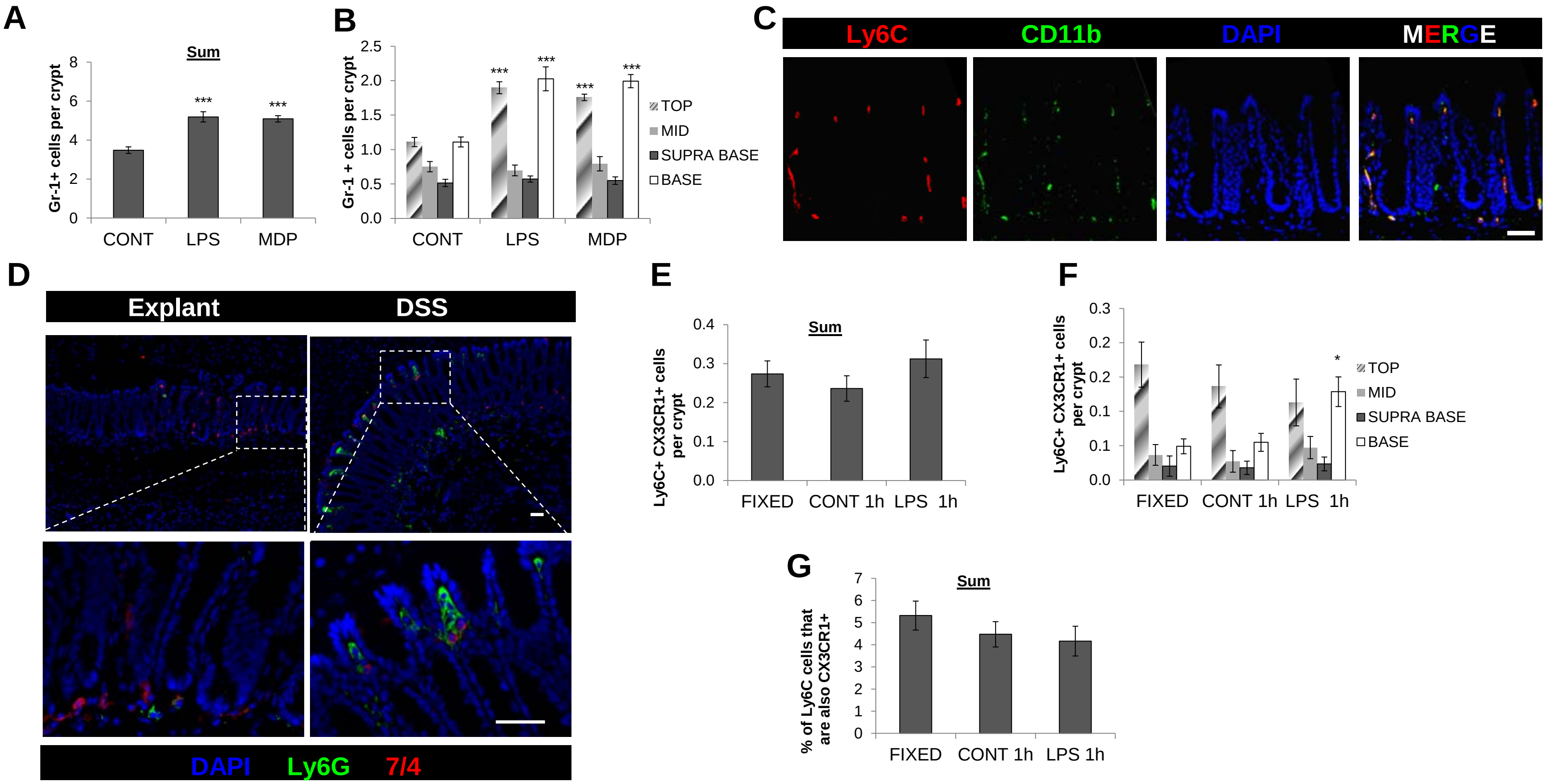

Supplementary Figure 2. Immune cell characterisation in mucosal explant culture

A Significant recruitment of Gr-1$^+$ cells into the mucosa and **B** significant recruitment of Gr1$^+$ cells to the top and base epithelium following 1 hour apical stimulation with LPS or MDP. Representative confocal images showing **C** Ly6C$^+$ (red) and cd11b$^+$ (green) co-staining in mucosal explants. **D** Low number of Ly6G$^+$ cells (green) in mucosal explant culture compared to colonic tissue from DSS-treated mice and lack of significant 7/4 antigen (red) co-localisation with Ly6G$^+$ (green) in mucosal explant culture. **E** No change in the number of Ly6C$^+$ CX3CR1$^+$ cells in the mucosa, but **F** a significant recruitment of Ly6C$^+$ CX3CR1$^+$ to the base epithelium following 1 hour apical stimulation with LPS (n=3, $^*P<0.05$). **G** Histogram showing a low percentage of Ly6C$^+$ cells are also CX3CR1GFP$^+$. Scale bar 50µm

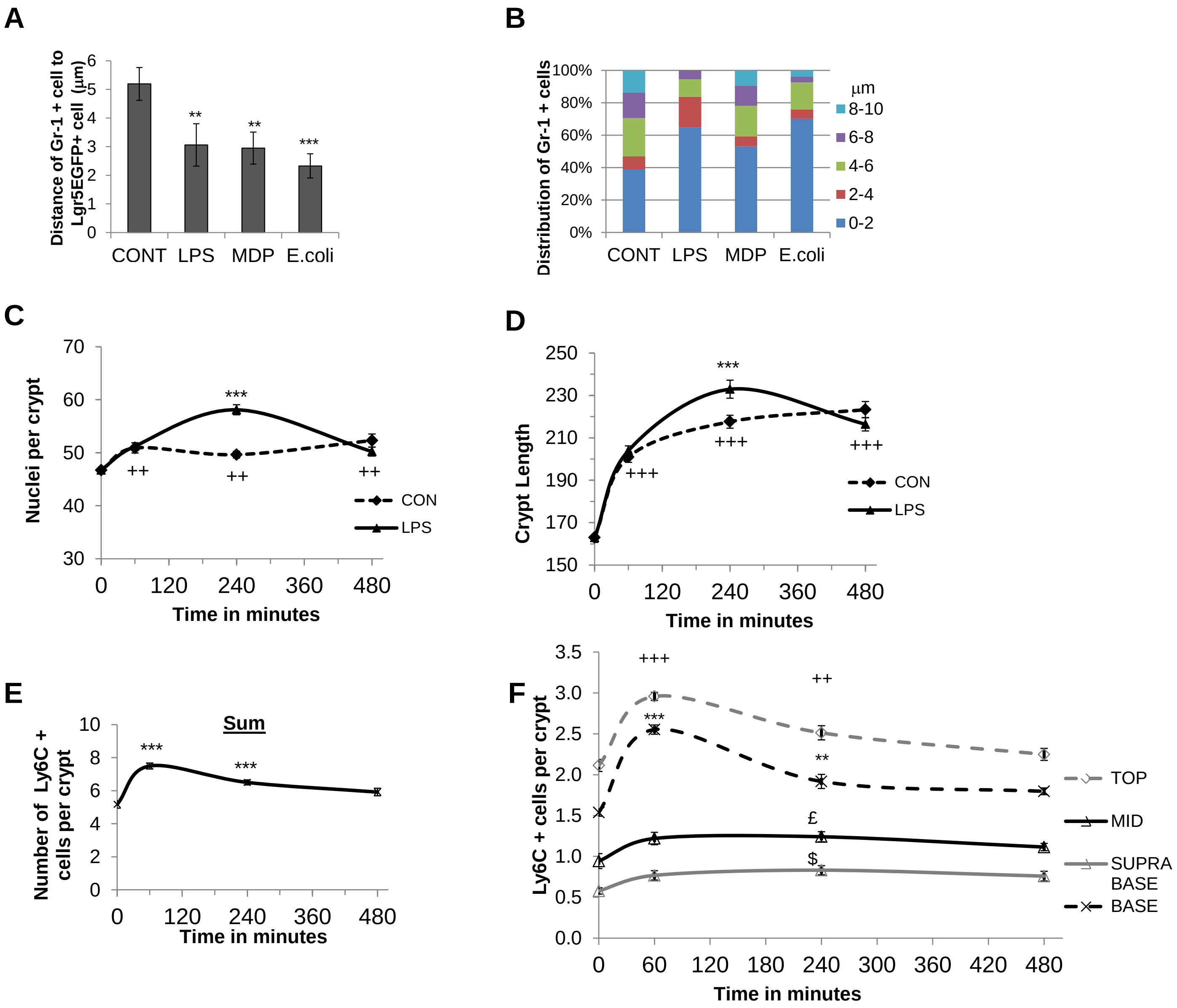

Supplementary Figure 3. Gr-1$^+$ cell distances to LGR5EGP$^+$ stem cells following microbial luminal input and crypt morphometry and Ly6C$^+$ cell distribution following 8 hours of mucosal explant culture with LPS.

A Gr-1$^+$ cells move closer to LGR5EGFP$^+$ stem cells following 1h apical treatment with LPS, MDP or *E. coli* (n=3, *P<0.01, **P<0.01, ***P<0.001). **B** Histogram shows the relative distribution of Gr-1$^+$ cells within a 10µm boundary of the LGR5EGFP$^+$ basal cell membrane. **C** Timecourse graph showing a significant increase in the number of nuclei per crypt in LPS-treated explants at 240mins compared to control-treated explants (n=3, ***P<0.001), also a significant increase in nuclei per crypt during mucosal explant culture (n=3, $^{++}$P<0.001). **D** Timecourse graph showing a significant increase in the crypt length in LPS-treated explants at 240 mins compared to control-treated explants (n=3, ***P<0.001), also a significant increase in crypt length during mucosal explant culture (n=3, $^{+++}$P<0.001). **E** A significant recruitment of Ly6C$^+$ cells to the mucosa following 60 and 240 mins apical stimulation with LPS (n=3, ***P<0.001) and a return to in vivo levels after 8h (480 mins). **F** Timecourse graph showing Ly6C$^+$ cell recruitment to specific zones of the crypt over 480 minutes. After 60 mins there is a significant increase in the number of Ly6C$^+$ cells at the base and top crypt epithelium (n=3, $^{***/+++}$P<0.001), compared to no treatment. After 240 mins the mid and supra base regions of the crypt also showed a significant increase in the number of Ly6C$^+$ cells (n=3, $^{£/\$}$ P<0.05). After 8 hours the distribution of Ly6C$^+$ cells along the crypt axis returned to control / baseline conditions.

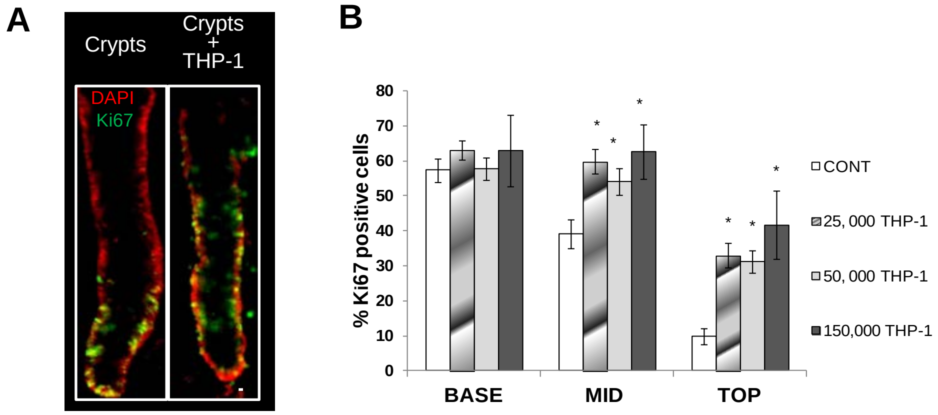

Supplementary Figure 4. THP-1 monocyte-like cells induce proliferation of cultured colonic human crypts. A Representative immunofluorescent images of human colonic crypts in co-culture with THP-1 cell line. **B** The number of $Ki67^{+}$ (green) positive nuclei were significantly increased ($^{*}P<0.05$) in the presence of THP-1 cells in the mid and top regions of the crypt. Data are represented as mean +/- SEM. Scale bar 10μm.
